# Identification of Human Kinin-Forming Enzyme Inhibitors from Medicinal Herbs

**DOI:** 10.3390/molecules26144126

**Published:** 2021-07-07

**Authors:** Hassan Madkhali, Amer Tarawneh, Zulfiqar Ali, Hoang V. Le, Stephen J. Cutler, Ikhlas A. Khan, Zia Shariat-Madar

**Affiliations:** 1 Department of Pharmacology, Faculty of Pharmacy, Prince Sattam Bin Abdulaziz University, Al-Kharj 16278, Saudi Arabia; h.madkhali@psau.edu.sa; 2 Department of Chemistry, Tafila Technical University, Tafila 66110, Jordan; amer.tarawneh@ttu.edu.jo; 3 National Center for Natural Products Research, School of Pharmacy, University of Mississippi, Oxford, MS 38677, USA; zulfiqar@olemiss.edu (Z.A.); ikhan@olemiss.edu (I.A.K.); 4 Department of BioMolecular Sciences, Division of Medicinal Chemistry, University of Mississippi, Oxford, MS 38677, USA; hle@olemiss.edu; 5 College of Pharmacy, University of South Carolina, Columbia, SC 29208, USA; sjcutler@cop.sc.edu; 6 Department of BioMolecular Sciences, Division of Pharmacology, University of Mississippi, Oxford, MS 38677, USA

**Keywords:** kinins, inflammation, angioedema

## Abstract

The goal of this study was to assess the pharmacological effects of black tea (*Camellia sinensis* var. assamica) water extract on human kinin-forming enzymes in vitro. Tea is a highly consumed beverage in the world. Factor XII (FXII, Hageman factor)-independent- and -dependent activation of prekallikrein to kallikrein leads to the liberation of bradykinin (BK) from high-molecular-weight kininogen (HK). The excessive BK production causes vascular endothelial and nonvascular smooth muscle cell permeability, leading to angioedema. The prevalence of angiotensin-converting enzyme inhibitor (ACEI)-induced angioedema appears to be through BK. Both histamine and BK are potent inflammatory mediators. However, the treatments for histamine-mediated angioedema are unsuitable for BK-mediated angioedema. We hypothesized that long-term consumption of tea would reduce bradykinin-dependent processes within the systemic and pulmonary vasculature, independent of the anti-inflammatory actions of polyphenols. A purified fraction of the black tea water extract inhibited both kallikrein and activated FXII. The black tea water extracts inhibited factor XII-induced cell migration and inhibited the production of kallikrein on the endothelial cell line. We compared the inhibitory effects of the black tea water extract and twenty-three well-known anti-inflammatory medicinal herbs, in inhibiting both kallikrein and FXII. Surprisingly, arjunglucoside II specifically inhibited the activated factor XII (FXIIa), but not the kallikrein and the activated factor XI. Taken together, the black tea water extract exerts its anti-inflammatory effects, in part, by inhibiting kallikrein and activated FXII, which are part of the plasma kallikrein–kinin system (KKS), and by decreasing BK production. The inhibition of kallikrein and activated FXII represents a unique polyphenol-independent anti-inflammatory mechanism of action for the black tea.

## 1. Introduction

There is widespread agreement that inflammation is an important transitional state at the crossroads of multiple pathophysiological conditions, including diabetic retinopathy [1], hereditary angioedema [2,3], and Alzheimer’s disease [4], all of which share many risk factors. Bradykinin, a metabolite of the plasma kallikrein–kinin system (KKS), is frequently amplified in hereditary angioedema [5]. The deficiency or non-functionality of the C1 inhibitor results in inflammation, and cutaneous and mucosa edema [6]. Studies in in vitro and in vivo models have shown the potential of the inhibitors of KKS as anti-inflammatory and anti-edema for hereditary angioedema, via the inhibition of kallikrein or the activated factor XII. Particularly, the modulation of inflammation is a potential therapeutic hereditary angioedema target.

**Figure 1:**
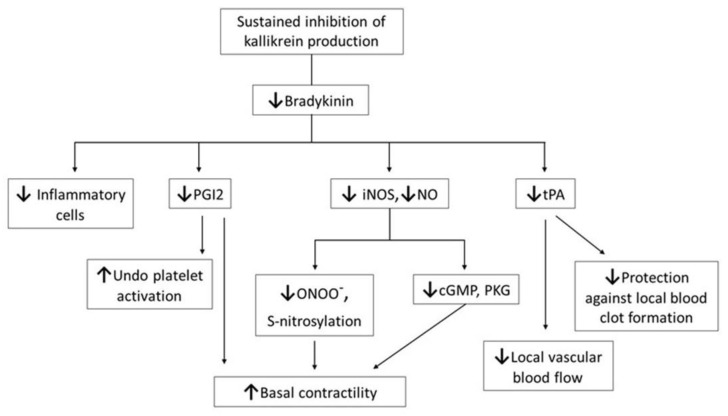
Schematic presentation showing that the inhibition of the plasma kallikrein–kinin action leads to loss of vascular protection. Over the long term, reduced BK (1) decreases production of iNOS with reduced NO generation; (2) decreases generation of PGI2 preventing its platelet aggregation inhibitory properties, and concomitant reduction in its vasodilating action; (3) reduces tissue plasminogen activator (tPA) with a consequent reduction in blood flow. Reduced levels of nitrosative leads to peroxynitrite formation, *S*-nitrosylation, and covalent protein thiol modifications of certain amino acid residues impairing basal contractility. However, reduced BK impairs cell permeability and tissue infiltration of inflammatory cells, which can slow tissue repair and healing.

The plasma KKS is one of the major control systems that helps circulation, as it causes the arteries in tissue(s) with low oxygen levels to open wider so that blood flows in. Bradykinin, a major effector of the plasma KKS, is produced by sequential activation of proteases and the cleavage of peptides derived from the substrate molecule high-molecular-weight kininogen (HK). Bradykinin acts in an endocrine fashion, via specific membrane-bound receptors, to signal a variety of physiological responses under normal conditions. For more detailed information on BK, please refer to the following review [7], and information on BK in amphibians [8]. Briefly, BK can impact blood vessel function via effects on both smooth muscle contractility and endothelial cells. As shown in Figure 1, BK regulates the following multiple physiological functions: (i) it protects the plasma cell membrane and vascular contractility, by maintaining the production of nitric oxide; (ii) it counteracts the constrictive property of thromboxane with potent hypertensive action, through the release of prostacyclin 2 (PGI2); (iii) it facilitates vasodilation via PGI2 [9]; (iv) it also inhibits platelet activation via PGI2; (v) it counteracts the prothrombotic property of the main effector, angiotensin II, of the renin–angiotensin system, through the release of tissue plasminogen activator (tPA) from endothelial cells; and (vi) it restores blood flow after local blood clot formation or ischemic insult, via tPA release [10]. BK has a complex regulatory influence on endothelial cells.

Sustained increases in the levels of kallikrein and FXII are observed across a broad spectrum of chronic diseases and insidious diseases. The elevation of kallikrein and activated FXII have been identified as principal triggers of pathology in numerous paradigms used to study chronic diseases, infection, or definable tissue damages, including local inflammation and ischemia. The C1 esterase inhibitor, a regulatory protease of the classical pathway of the complement system, is a major inhibitor of both kallikrein and activated FXII. Notably, patients with C1 inhibitor deficiency, a hereditary angioedema, exhibit uncontrolled inflammation, due to the elevation of kallikrein activity [11], and subsequent overproduction and accumulation of BK [12], which is the major mediator of angioedema. Nevertheless, BK causes resistant blood vessels to be partially dilated under basal conditions. Gain-of-function mutations of the FXII gene with the normal C1 inhibitor also cause a hereditary angioedema-like phenotype [13]. Mice with a deficiency of FXII clearly show that all of the contact-stimulated plasma BK is provided by the FXII-dependent pathway in the absence of factor XI [14]. Notably, loss-of-function models of kininogens, BK precursors, exhibit reduced angiogenesis and reduced inflammation [15]. Thus, plasma BK level is an important consideration in the evaluation of FXII/kallikrein-dependent dysfunction under physiological and pathophysiological conditions.

During an inflammatory response, the plasma KKS plays a role in inflammatory responses, including cell permeability, vascular relaxation, and angiogenesis. Although genomic analysis is not done to quantify the penetrance of hereditary angioedema worldwide, hereditary angioedema is more prevalent in Europe and North America [16] than the estimated prevalence in Asia, particularly in Japan [17], and with the exception of China [18].

Medicinal herbs have been used to treat diseases related to inflammation for centuries. We searched for an ingredient that is capable of inhibiting plasma kinin-forming enzymes. Phytochemicals exhibit great anti-inflammatory potential [19] in a growing body of preclinical research, through numerous strategies, including modulating cytokines and oxidants. Tea consumption is associated with a lower risk of death from coronary heart disease and stroke [20]. An inverse association is found between tea intake and the severity of aortic atherosclerosis [21]. Notably, KKS appears to have a role in aortic endothelial cells [22]. The countries with a high per capita tea consumption [23] report low frequency and severity of hereditary angioedema attack among their people. Similarly to many other plants and herbs, black tea possesses an array of bioactive compounds, with robust pharmacological values [24]. We hypothesized that long-term consumption of black tea would reduce plasma kinin-forming enzymes’ activity within the systemic and pulmonary vasculature, independent of the anti-inflammatory actions of polyphenols. To address this hypothesis, we determined and characterized the inhibitory effects of the water crude extract of tea and its major bioactive compounds on plasma kinin-forming enzymes. In addition, we also screened the inhibitory effects of herbs, and phytochemicals with potential clinical applications, in modulating kinin-forming enzymes or the synthesis of new anti-edema and anti-inflammatory agents that take phytochemicals as lead compounds.

## 2. Results

### 2.1. Activated FXII and Kallikrein Activities and Assays

**Figure 2:**
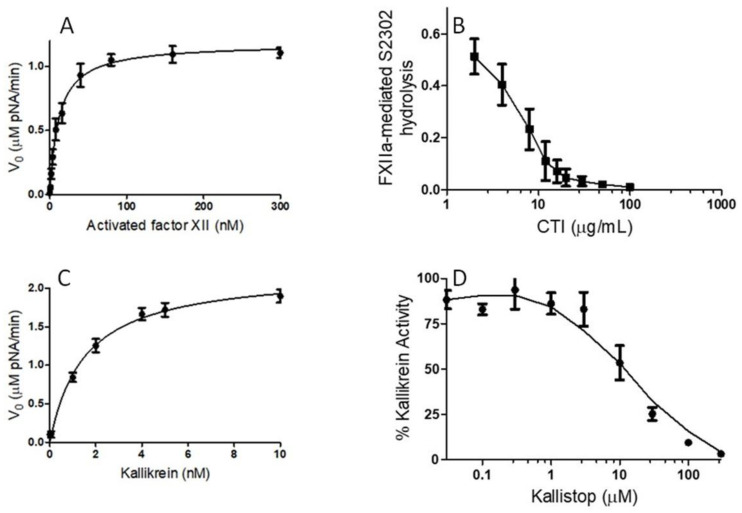
Characterization of human blood coagulation activated FXII (FXIIa) and kallikrein. (**A**) Chromogenic substrate assay for FXIIa activity. Varying concentrations of FXIIa (1–300 nM) were incubated with S2302 (0.5 mM). FXIIa activity was measured by monitoring the rate of S2302 hydrolysis. Changes in OD 405 nm/minute were measured on a plate reader and converted to pNA generated per minute. (**B**) Effect of corn trypsin inhibitor on FXIIa. FXIIa incubated in the absence or presence of increasing concentrations of corn trypsin inhibitor (1–100 μg/mL). (**C**) Chromogenic substrate assay for kallikrein activity. Varying concentrations of kallikrein (1–10 nM) were incubated with S2302 (0.5 mM). Kallikrein activity was measured by monitoring the rate of S2302 hydrolysis. (**D**) Effect of kallistop on kallikrein. Kallikrein incubated in the absence or presence of increasing concentrations of kallistop (0.05–300 μM). Data are the means of three separate experiments.

In order to study whether black tea was able to protect against the over-production of BK, we used two chromogenic activity assays that are commonly used to measure activated FXII and kallikrein activities that function as central components of the plasma KKS. To determine the optimum activated FXII concentration, with a constant S2302 concentration, increasing amounts of activated FXII (1–300 nM) were used in the activated FXII reaction. Figure 2A indicated that the activated FXII-mediated S2302 (0.5 mM) hydrolysis is concentration-dependent. The optimum concentration of activated FXII, to achieve a good signal-to-noise ratio, was found to be approximately 17 nM. Saturation was achieved between 20 and 300 nM. To determine the optimum amount of S2302 for a fixed concentration of activated FXII (17 nM), increasing concentrations of S2302 (0.1–2 mM) were used to quantify the paranitroanalide released from S2302 by activated FXII. The K_m_ was 0.4 ± 0.15 mM (data not shown). Corn trypsin inhibitor (CTI) is a commercially available selective inhibitor of activated factor XII. To determine whether herb extracts, including black tea water extract, or their bioactive compounds could yield a more potent inhibitor of activated factor XII than the CTI, activated factor XII (17 nM) was added to the reaction buffer, which contained increasing concentrations of CTI (1–1000 μg/mL), along with S2302 (0.4 mM). CTI inhibited the activated FXII, with an IC_50_ value of 6.5 ± 1.3 μg/mL (Figure 2B). CTI was used as a positive control.

To achieve an optimum concentration of kallikrein for the inhibition study of herb extracts and their bioactive molecules, various concentrations of kallikrein were used, in the presence of a fixed concentration of S2302 (0.5 mM). Figure 2C revealed that increasing concentrations of kallikrein led to a concentration-dependent increase in S2302 hydrolysis. Kallikrein was characterized under pH 7.4. The optimum concentration of kallikrein, to achieve a good signal-to-noise ratio, was found to be 2 nM. To quantitatively determine the activity of kallikrein (2 nM), we evaluated the Michaelis constant for the degradation of S2302. The K_m_ was determined to be 0.35 ± 0.12 mM (data not shown). Kallistop, a selective inhibitor of kallikrein, was used as a positive control for screening herb extracts and bioactive molecules for potential kallikrein or activated FXII inhibitors. Increasing concentrations of kallistop caused a concentration-dependent decrease in kallikrein activity (Figure 2D). Kallistop inhibited kallikrein, with an IC_50_ value of 10 ± 2.1 μM.

### 2.2. The Effects of the Extracts of Tea and Herbal Medicines on Human Plasma Kinin-Forming Enzymes

**Figure 3:**
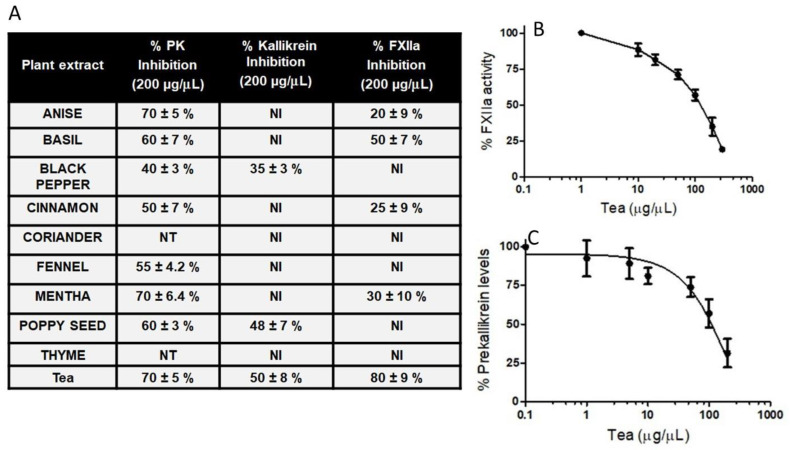
Effect of black tea water extract on activated FXII, plasma kallikrein activity, or the activation of prekallikrein (PK). Increasing concentrations of black tea water extract were incubated with activated FXII (**A**,**B**), kallikrein (**A**), or a mixture of high-molecular-weight kininogen (HK) and PK (HK/PK) on HAEVC (**C**) and S2302 (0.5 mM) for 1 h at 37 °C. For all panels, data are means for three independent experiments. NI; denotes no inhibition. NT; not tested.

To test the hypothesis that herbs could modulate BK-induced angioedema, we assessed herbs and tea for their ability to inhibit kallikrein and activated FXII, which are involved in the liberation of BK from high-molecular-weight kininogen. The most common herbs were selected, because they would be routinely added at the beginning or during the cooking of food or tea. Among thirty different herbs, those with anti-inflammatory properties were chosen (Figure 3). The water extracts of ten herbs showed concentration-dependent inhibition on both kallikrein and activated FXII. A ceiling effect was observed for the herb water extract effect at 200 μg/μL. The 200 μg/μL concentration of the tea’s water extract is within the recommended guideline in brewed teas [25], without causing any hepatotoxicity. Figure 3A shows the hydrolysis of substrates by activated FXIIa, kallikrein, and the activation of prekallikrein on HPAECs, in the absence and presence of various herb water extracts, as described under Methods. As expected, not all herb extracts were capable of inhibiting the activated FXIIa, kallikrein, and the activation of prekallikrein on HPAECs. Eight extracts (200 μg/μL each) inhibited the activation of prekallikrein to kallikrein on HPAECs (Figure 3A). Among the ten extracts, black pepper (200 μg/μL), poppy seed (200 μg/μL), and tea water extracts inhibited kallikrein-mediated hydrolysis of S2302, by 35%, 48%, and 50%, respectively. Of these herb extracts, tea inhibited the activated FXIIa-mediated hydrolysis of S2302 by 80%, and with an IC_50_ of 98 ± 4.2 μg/μL (Figure 3, Panel B). Notably, tea water extract was less potent than CTI (Figure 2B).

Using a prekallikrein activation assay on HPAECs, we determined the IC_50_ values of ten herb extracts (data not shown). The results showed that fennel was ineffective in inhibiting prekallikrein activation on HPEACs. Anis was the most potent, with an IC_50_ value of about 26 μg/μL. Black pepper, cinnamon, coriander, poppy seed, and thyme were approximately five times less potent than anise. Mentha inhibited prekallikrein activation, with an IC_50_ of 80 μg/μL. Tea water extracts inhibited prekallikrein activation, with an IC_50_ of 96 ± 6.1 μg/μL (Figure 3C). Neither extract was as potent as anise extract in inhibiting prekallikrein activation. None of the herb extracts were more potent than kallistop (Figure 2D).

### 2.3. The Effects of the Major Tea Biomolecules on the Kallikrein-Mediated and the Activated Factor XII-Mediated Hydrolysis of Substrate

Polyphenol molecules, purine alkaloids, and tannins are widely distributed within the plant kingdom, including tea. The major tea biomolecules consist of theophylline, gallic acid, and eight catechins. We sought to determine the effect of these bioactive molecules on the FXIIa-mediated hydrolysis of S2302. Unexpectedly, (−)-epigallocatechin-3-gallate (EGCG), (−)-epigallocatechin (EGC), (−)-epicatechin-3-gallate (ECG), (−)-epicatechin (EC), quercetin, theophylline, and caffeine were ineffective in directly inhibiting the hydrolysis of S2302 (0.4 mM), by activated FXII (17 nM), at a concentration between 100 and 1000 μM (data not shown). Collectively, these findings suggested that tea water extract contains uncharacterized compound(s) that were, in fact, capable of inhibiting the activated FXII (Figure 3B).

**Figure 4:**
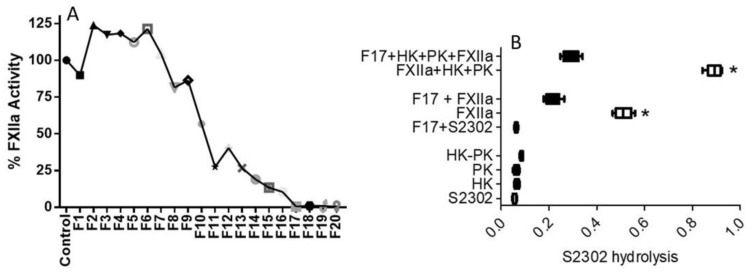
Showing the effect of biologically active compounds in tea on activated FXII (**A**) and FXIIa-induced prekallikrein (PK) activation (**B**) after HPLC separation. (**A**) Profile of anti-FXIIa activity in HPLC fractions. Each symbol indicates pNA formation in the presence of FXIIa (17 nM) supplemented with S2302 (0.5 mM). (**B**) pNA generation in HPLC fraction 17 supplemented with S2302 in the absence or presence of FXIIa or FXIIa and a mixture of HK and PK. The horizontal bars indicate means for each group ±1 SD. For all panels, results represent means for three runs (* *p* < 0.05 compared to F17-treated FXIIa).

The tea water extract that had anti-FXIIa activity was further purified using HPLC (see Materials and Methods). About 20 HPLC fractions (75 μL each) were collected and tested for anti-FXIIa activity. All the fractions’ activities were measured against the activated FXIIa. Among the 20 fractions, only fourteen HPLC fractions exhibited anti-FXIIa activity, while the remaining fractions showed no anti-FXIIa activity. Of those fourteen, fraction 11 (F11) through to fraction 20 (F20) were particularly effective in inhibiting FXIIa, by at least 75% (Figure 4A). These results are in line with the concentration-response data that showed full inhibition of activated FXIIa at a lower concentration of 50 μg/mL. Inhibition of FXII autoactivation was also determined in parallel, and essentially matched the inhibition of the activated FXIIa-mediated hydrolysis of the substrate (Figure 4B). Prekallikrein, HK, S2302 alone, or a combination of prekallikrein and HK, did not result in hydrolysis of the substrate. Activated FXII hydrolyzed S2302, whereas one HPLC fraction (F17) particularly inhibited the hydrolysis of S2302 by activated FXII (Figure 4B). F17 also inhibited FXII autoactivation. The hydrolysis of S2302 was significantly increased in the presence of a combination of HK, PK, and activated FXIIa, while its hydrolysis was inhibited by fraction 17.

### 2.4. Anti-Inflammatory Potential of Ayurvedic Medicine and Its Major Bioactive Compounds on FXIIa-Mediated Hydrolysis of Substrate

**Figure 5:**
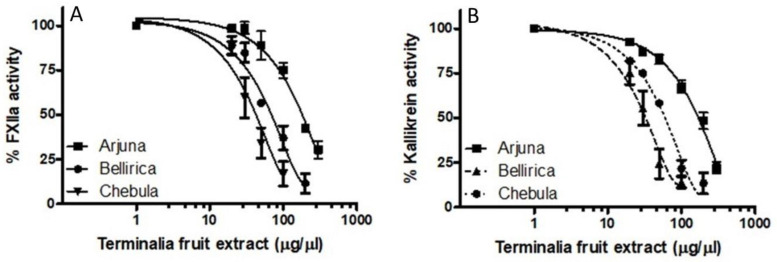
Effect of *Terminalia* fruit extract on plasma kallikrein activity. Increasing concentrations (1–500 μg/μL) of *Terminalia* fruit extract were incubated with activated factor XII (17 nM), (**A**) kallikrein (**B**) and S2302 for 1 h at 37 °C. For all panels, results represent means for three runs.

Given the striking effects of tea water extract on activated FXII and prekallikrein activation, we asked whether our finding reflected a more generalized phenomenon. Similarly to tea, *Terminalia* plants are also widely used, with numerous therapeutic properties, including anti-inflammatory properties, for traditional medicinal purposes worldwide [26]. We explored the effects of Ayurvedic medicine (*Terminalia arjuna*, *Terminalia bellerica*, *Terminalia chebula*) on FXIIa activity, and compared it with that of tea. The results showed that *Terminalia arjuna* fruit extract inhibited the FXII-mediated S2302 hydrolysis assay, with an IC_50_ value of 190 ± 7.5 μg/μL (Figure 5A). In a head-to-head comparison in the same biochemical assay, *Terminalia bellerica* fruit extract had an IC_50_ of 70 ± 8.4 μg/μL, whereas *Terminalia chebula* fruit extract had an IC_50_ of 54 ± 7.5 μg/μL (Figure 5A). However, the inhibitory potential of the bark extracts of the aforementioned Ayurvedic medicine was insignificant in inhibiting FXII (data not shown). Uncharacterized inhibitors of activated FXII are also found in various *Terminalia*, although the potency of the inhibitors varied. Comparing tea water extract to *Terminalia* fruit extract, both the tea and *Terminalia* extracts showed essentially BK-modulating properties that are capable of inhibiting activated FXII.

Studies next determined the effects of *Terminalia* on kallikrein function. *Terminalia arjuna* fruit extract inhibited the kallikrein-mediated hydrolysis of S2302, with an IC_50_ value of 200 ± 6.6 μg/μL (Figure 5B). In a head-to-head comparison in the same biochemical assay, *Terminalia bellerica* fruit extract had an IC_50_ of 50 ± 9.4 μg/μL, whereas *Terminalia chebula* fruit extract had an IC_50_ of 80 ± 4.0 μg/μL (Figure 5B).

The importance of *Terminalia arjuna*, *Terminalia bellerica*, and *Terminalia chebula* was further elucidated by profiling the potential inhibitory effects of the major bioactive compounds of *Terminalia* on FXIIa. Arjunic acid, terminolic acid, arjungenin, arjunglucoside I, chebuloside II, arjunolic acid, corilagin, gallic acid, gallic acid methyl ester, shikimic acid, ellagic acid, chebulagic acid, chebulinic acid, as well as 2-, 3-, 6-tri-*O*-galloyl-β-d-glucose; 1-, 2-, 3-, 6-tetra-*O*-galloyl-β-d-glucose; and 1-, 2-, 3-, 4-, 6-penta-*O*-galloyl-β-d-glucose were ineffective in inhibiting activated FXII at a concentrations between 10 μM and 300 μM (data not shown). Surprisingly, arjunglucoside II inhibited the activated factor XII, with an IC_50_ value of 300 μM. Although this IC_50_ value is significantly higher than the in vitro potency benchmark (IC_50_ < 100 nM in biochemical assays), a nearly complete inhibition of the activated FXII activity at saturating concentrations of arjunglucoside II was observed. The fact that the remaining compounds did not display any inhibitory activity indicates that FXII is highly selective in its choice of reactants. Thus, arjunglucoside II can be considered to be a potentially useful inhibitor of activated FXII.

Next, we determined the selectivity of the major bioactive compounds of *Terminalia* in activity assays, against FXIa and kallikrein. The active sites of activated FXII, activated FXI, and kallikrein have huge similarities. Among all the aforementioned bioactive compounds of *Terminalia*, none of the compounds inhibited kallikrein. chebuloside II, corilagin, and gallic acid inhibited activated FXI by 30% at a concentration of 300 μM. However, arjunglucoside II did not inhibit either activated FXI or kallikrein. Collectively, arjunglucoside II selectively inhibited the activated factor XII, but did not cause the inhibition of kallikrein or activated FXI.

**Figure 6:**
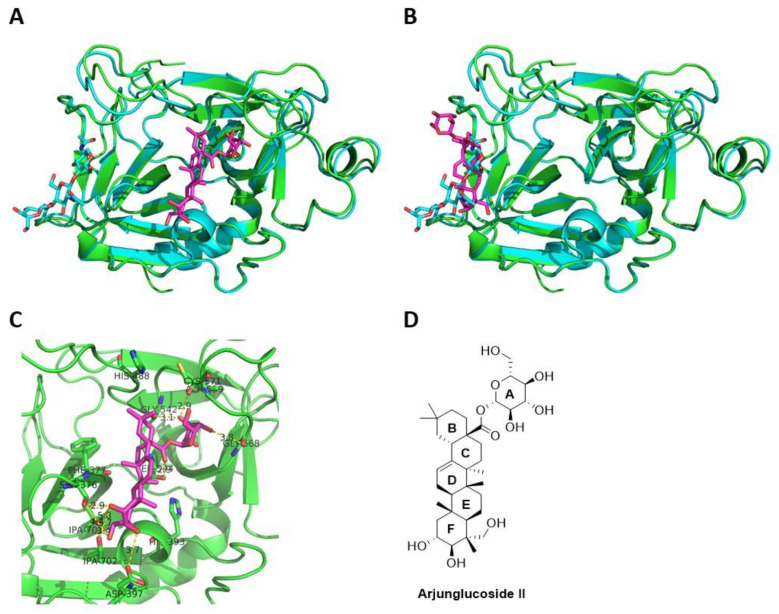
Molecular docking studies of arjunglucoside II in FXII. (**A**) Docked arjunglucoside II (magenta) in the active site of the deglycosylated catalytic domain of factor XII (green, PDB: 4XDE), superposed with the glycosylated catalytic domain of factor XII (cyan, PDB: 4XE4); (**B**) docked arjunglucoside II (magenta) in the glycosylated site of the deglycosylated catalytic domain of factor XII (green, PDB: 4XDE), superposed with the glycosylated catalytic domain of factor XII (cyan, PDB: 4XE4); (**C**) important interactions between docked arjunglucoside II (magenta) and nearby residues in the active site of the deglycosylated catalytic domain of factor XII (green, PDB: 4XDE); (**D**) structure of arjunglucoside II.

Computational docking studies next allowed us to explore the binding modes of arjunglucoside II in factor XII. We performed molecular docking studies, using GOLD [27] of arjunglucoside II, on the crystal structure of the deglycosylated catalytic domain of factor XII (PDB: 4XDE), and discovered that arjunglucoside II would bind really well to both the active site (Figure 6A) and the glycosylated site (Figure 6B) of the catalytic domain of FXII. The best poses of arjunglucoside II from docking studies at both the active site and the glycosylated site were analyzed, and important interactions with nearby residues were identified. For example, Figure 6C shows important interactions between the best docking pose of arjunglucoside II and the nearby residues in the active site of the deglycosylated catalytic domain of factor XII. The hydroxyl groups on the sugar ring A strongly interact with Gly542 and Gly568 through hydrogen bonding, while the hydroxyl groups on ring F strongly interact with Ser376 and Asp397 through hydrogen bonding. Rings B, C, D, and E only interact with the active site through hydrophobic interactions. Overall, molecular docking studies substantiated the above experimental studies. Arjunglucoside II appears to be a suitable lead compound for the further development of inhibitors of activated factor XII against BK-induced angioedema.

### 2.5. The Tea Water Extract Interferes with FXII-Induced Cell Migration In Vitro

**Figure 7:**
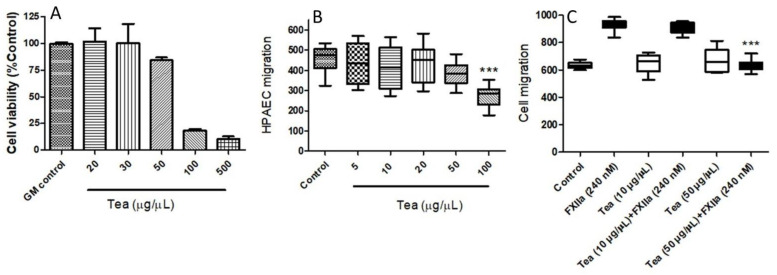
**Showing cell viability and cell migration.** (**A**) Effect of tea water extract on HPAEC viability. MTT assay was performed to measure cell viability (by percent) in response to tea water extract treatment. HPAECs treated with tea water extract using the MTT assay. The complete growth medium (GM) was used as a control. Data represent the mean ± SEM of three experiments (*** *p* < 0.01 compared to untreated control). (**B**) Effect of tea water extract on HPAEC migration in the absence of activated FXII. Cells were seeded, cultured, and allowed to grow to near confluence. Confluent monolayers were carefully wounded and the cellular debris was gently washed away with PBS. Cells were treated with increasing concentrations of tea water extracts. Each bar represents nine runs. Statistical significance was measured with the two-way ANOVA. *** *p* < 0.001 compared to control. (**C**) Effect of tea water extract on HPAEC migration in the presence of activated FXII (FXIIa). Cells were seeded, cultured, and allowed to grow to near confluence. Confluent monolayers were carefully wounded and the cellular debris was gently washed away with PBS. Cells were treated with various concentrations of tea water extracts in the presence of FXIIa. For panel C, each bar represents nine runs (*** *p* < 0.001 compared to FXIIa).

To further substantiate whether the relative inhibition of FXIIa activity could impact the overall FXII activity profile, the effects of inhibitors on FXII-induced cell migration were explored. We initially determined cell viability using the MTT assay. HPAECs were treated with various concentrations of tea water extracts (20–500 μg/μL). Figure 7A reveals that increasing concentrations of tea water extracts induced a concentration-dependent increase in toxicity, by the decrease in cell viability. The cell viability assay, along with a phenotypic observation of apoptosis under microscopy, suggested that the 50 μg/μL tea water extracts did not induce cell apoptosis. The tea water extracts at concentrations of 100 μg/μL and 500 μg/μL had lethal effects after 24 h of culture. Therefore, the 10 μg/μL and 50 μg/μL tea water extracts were the concentrations chosen for the experiments that followed.

Activated FXII has been demonstrated to be a contributor to angiogenesis and tissue injury repair [28]. Next, we examined cell migration in response to the mechanical scratch wound, in the absence or presence of increasing concentrations of tea water extracts, ranging from 5 to 100 μg/μL. Lower concentrations of tea water extract (<50 μg/μL) did not affect cell migration and morphology significantly, whereas they significantly inhibited HPAECs migration at 100 μg/μL (Figure 7B). To quantify the effects of various concentrations of tea water extract on FXIIa-induced cell migration, the migration of cells to the open wound area after 24 h was determined. Our data demonstrated that, while no migration inhibition was observed at 10 μg/μL, the treatment with tea water extract (50 μg/μL) caused a significant inhibition of FXIIa (240 nM)-induced HPAEC migration (Figure 7C).

### 2.6. Tea Water Extract Inhibits the Release of BK from High-Molecular-Weight Kininogen by Kallikrein

**Figure 8:**
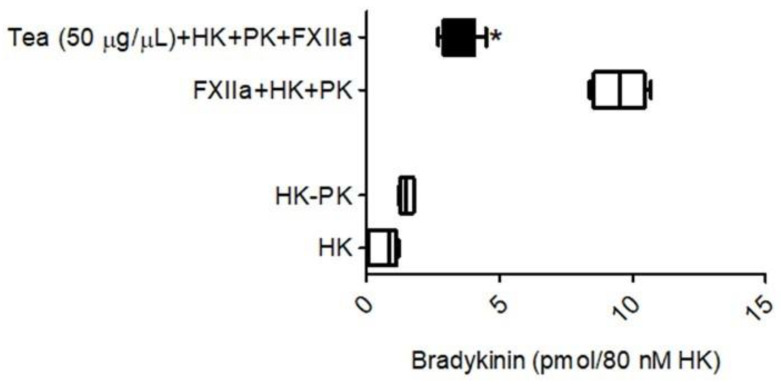
Showing the effect of tea water extract on BK production. Hydrolysis of high-molecular-weight kininogen (HK, 80 nM) was studied by following the changes in the amounts of product, BK in the absence or presence of the activated factor XII (FXIIa) and prekallikrein (PK, 80 nM). Each bar represents nine runs. Statistical significance was measured with the two-way ANOVA. * *p* < 0.05 compared to FXIIa + HK + PK.

The investigations proceeded next to determine whether the tea water extract would prevent the activation of prekallikrein by activated FXIIa, leading to a reduction in BK from high-molecular-weight kininogen. As shown in Figure 8, BK (9.7 × 10^6^ pmol) was released into the incubation buffer when the complex of HK/PK was added to activated FXII (4 nM). However, tea water extract (50 μg/μL) inhibited the activated FXII-mediated kallikrein generation, resulting in a decrease in the liberation of BK (4.2 × 10^6^ pmol) into the incubation buffer (Figure 8). The amount of BK generation was 50 percent lower in the presence of tea water extract than in the control. No BK was detected in the absence of added activated FXII.

## 3. Discussion

The inactivation of kallikrein is impaired in hereditary angioedema (HAE) patients [29]. Skin swellings (70%), life-threatening laryngeal edema (1%), and abdominal pain attacks (54%) are frequently reported in patients with HAE [30,31]. HAE can be life-threatening, due to severe upper airway obstruction [32,33]. Patients with HAE do not respond to epinephrine, antihistamines, or corticosteroids. Several studies have clearly shown that BK serves as a major pro-inflammatory mediator of the symptoms of C1 inhibitor deficiency [34,35]. Recent evidence indicates that a defective control of the plasma BK-forming cascade can cause vascular defects [36,37].

The kallikrein–kinin system (KKS, contact system, or intrinsic pathway) is composed of three zymogens (PK, factor XI, factor XII) and one cofactor (HK, BK precursor), which are involved in the coagulation pathway and kallikrein–kinin forming pathway, in response to vascular injury, abnormality, and infection. While it is the rate-limiting protease in the two pathways mentioned above, FXIIa appears to catalyze different activities depending on its stimulation amplitude state. The C1 inhibitor forms 1:1 stoichiometric complexes with factors XII [38], XI [39], and kallikrein [40]. There is evidence that decreased levels of the C1 inhibitor are associated with sepsis [41] (but not in neonatal sepsis [42]), acute myocardial infarction [43], lymphoma [44,45], and lymphoproliferative disorders [46]. These clinical observations do provide strong evidence that low C1 inhibitor levels in these subjects, due to a marked consumption of C1 inhibitor, could lead to an excessive or uncontrolled release of proinflammatory BK via the FXIIa-mediated pathway. This is particularly relevant in view of the recent evidence from preclinical and clinical studies with bradykinin type 2 receptor inhibitors, which has shown that BK contributes to the pathogenesis of diseases of the cardiovascular system and of inflammatory diseases that lead to pain. On the basis of a double-blind randomized multicenter study, bradykinin B_2_ receptor antagonist was ineffective in improving the outcomes in patients with systemic inflammatory response syndrome and sepsis. The marked decrease in the serum levels of the C1 inhibitor can result in FXIIa and kallikrein half-life prolongation, which would produce intense potentiation of HAE, but can also cause consumption of the complement components. Moreover, the activation of FXII has been reported in various types of human diseases, including Alzheimer’s disease [47], rheumatoid arthritis [48], sepsis, Porphyromonas gingivalis [49,50], bacterial infection [51,52], parasite infection [53], virus-induced endothelial cell activation [54], and HSV1 [55]. Thus, the increased level of FXII plays a role in the pathogenesis of numerous diseases by triggering BK generation and thrombin formation.

A large number of herbal products and their constituents have shown inhibitory activity related to inflammation, cancer, and angiogenesis. Green teas are known for their potential health benefits, which has been confirmed with preclinical and epidemiological studies related to various diseases [56,57], both bacterial [58] and viral [59] infections, as well as their neuroprotective properties [60]. Regardless of the types of tea, their components are known to be associated with some amount of bioactivity. Tea polyphenols are found in foods such as biscuits [61] and noodles [62]. It is known that polyphenolic dietary supplementation influences cognitive deficits in individuals of advanced age [60].

In this present study, the interaction between tea water extract and the activated FXII and kallikrein were investigated. Here, we provided evidence that tea water extract inhibited these enzymes in vitro, to different extents. In addition, the tea water extract also inhibited activated FXII-induced cell migration at concentrations that were lower than those causing cytotoxicity. Tea water extract alone did not cause cell migration or inhibit cell migration. In contrast to our findings, evidence indicates that (−)-epicatechin gallate (ECG), an active ingredient extracted from tea, is capable of inhibiting the migration of vascular smooth muscle cells [63], and green tea water extract is associated with actin modeling and cell migration [64]. One plausible explanation could be that the amount of ECG in our black tea water extract preparation was not high enough to inhibit HPAEC migration. Nevertheless, tea water extract in amounts greater than 100 μg slowed cell migration (Figure 7B).

The major polyphenols in tea failed to inhibit activated FXII. Surprisingly, among the major bioactive compounds of *Terminalia*, arjunglucoside II inhibited the activated FXII. Molecular docking studies showed the intermolecular interactions between FXII and arjunglucoside II. This bioactive molecule docked well in the substrate-binding site and glycosylated site of the catalytic domain of the activated FXII. Arjunglucoside II, as a novel lead compound, which selectively targets FXII, shows promise as a treatment for diseases that are associated with overactive FXII, and may lead to the development of a new drug.

## 4. Materials and Methods

### 4.1. Materials

Single-chain HK was purchased from Enzyme Research Laboratories (South Bend, IN, USA). S2302; BIOPHEN CS-31 (02) was purchased from Aniara (Mason, OH, USA). HK was more than 95% of a single band of 110 kD on both nonreduced and reduced SDS electrophoresis. Kallikrein, prekallikrein, FXIa, α-FXIIa, and β-fXIIa were purchased from Enzyme Research Laboratories (South Bend, IN, USA). Prekallikrein and FXII were more than 95% pure. FXII, corn trypsin inhibitor (CTI), and all other coagulation proteins were purchased from Haematologic Technologies (Essex Junction, VT, USA). Kallistop, a 4-amidinoanilide derivative of phenyl-2-aminobutyric acid, a selective inhibitor of kallikrein, was purchased from American Diagnostica Inc. (Stamford, CT, USA). Bovine serum albumin (BSA) was purchased from EMD Chemicals (Gibbstown, NJ, USA). Reagents with more than 95% purity were purchased from Sigma (St. Louis, MO, USA).

### 4.2. Cell Cultures

Human pulmonary artery endothelial cell (HPAEC) was obtained and cultured in growth medium 200 supplemented with low serum growth supplement according to the manufacturer’s protocol (Invitrogen, NY, USA) into 96-well culture plates and incubated in a CO_2_ controlled incubator (5% CO_2_) at 37 °C overnight.

### 4.3. Extraction Protocol and Chromatographic Condition

Commercially available black tea and individual herbs were used in this study. Each herb (4 g) and the black tea leaves (4 g) were powdered and used for extraction. Each herb and tea powder was weighed separately and added to 10 mL of boiled deionized water and allowed to simmer for 30 min. Each solution was filtered through a Whatman number 1 filter paper and centrifuged at 10,000× *g* for 10 min. The supernatant of tea water extract and each herb’s extracts were collected and used for the kinetic studies and HPLC. Each extraction was conducted in triplicate and tea water extract was put for HPLC analysis in duplicate.

Reversed-phase high-performance liquid chromatography (RP-HPLC) was carried out to purify the bioactive compounds in the tea water extract. Tea water extracts were centrifuged and the supernatant (25 μL) was injected into the column. Tea constituents were separated at a flow rate of 0.5 mL/min during the entire analytical run and the column temperature was set at 38 °C.

### 4.4. Prekallikrein Activation on HPAEC

Kinetic studies were performed on confluent HPAEC (4 × 10^4^ cells/well) in their second to sixth passage, in 96-well microtiter plates. Within 24 h of reaching confluence, the cells were washed three times in HEPES-Tyrode buffer (135 mM NaCl, 2.7 mM KCl, 11.9 mM NaHCO_3_, 0.36 mM NaH_2_PO_4_, 14.7 mM HEPES, pH 7.35) containing 100 mg/mL dextrose and 0.1% gelatin (HEPES-Tyrode gelatin buffer). Cells were incubated with 20 nM HK or BK-free HK at 37 °C for 60 min in the same buffer. After the removal of unbound HK, the cells were incubated with 20 nM PK alone or in the presence of various concentrations of the plant extract (0.001 mg to 500 mg) or bioactive compounds (0.001 mM to 500 mM) in the same buffer for 1 h at 37 °C. The HPAEC were then washed followed by adding 100 μL of 0.4 mM HD-Phe-Pro-Arg-p-aranitroanilide (S2302) (DiaPharma; Franklin, OH, USA) and incubated for 1 h at 37 °C. The activity of kallikrein generated was measured at 405 nm.

### 4.5. Chromogenic Assays

The following assays were performed in 96-well flat-bottom microtiter plates (Corning Incorporated, Corning, NY, USA) at 37 °C in HEPES-carbonated buffer (14.7 mM HEPES, 137 mM NaCl, 3 mM KCl, 12 mM NaHCO_3_, 5.5 mM glucose, 0.1% gelatin, 2 mM CaCl_2_, 1 mM MgCl_2_, pH 7.1). While the chromogenic bioassay technique is an easy assay to identify the inhibitors of the activated FXII and kallikrein, the color of plant extracts was interfering with the resultant absorbance. An additional blank was added to our bioassay to analyze the contribution of background absorbance of plant extract color to the signal. This background color control was run alongside individual herb extract in every assay run. All coagulation protein concentrations were finalized, and the rate of substrate hydrolysis was recorded at 405 nm on a BioTek ELx800 absorbance microplate spectrophotometer (Biotek, Winooski, VT, USA). Data were analyzed using the Graphpad Prism software with linear regression analysis unless indicated otherwise (GraphPad Prism, GraphPad software, Inc., San Diego, CA, USA).

### 4.6. Kallikrein Substrate Cleavage

Kallikrein (20 nM) was incubated with varying concentrations of herb extracts, each fraction obtained in the purification by HPLC, bioactive molecules, or kallistop, a selective inhibitor of kallikrein, as a positive control for 10 min before adding S2302 (0.3 mM) Aniara (Mason, OH, USA).

### 4.7. FXIIa Small Peptide Substrate Cleavage

FXIIa (10 nM) was incubated with varying concentrations of tea water extract, in the absence or presence of each fraction obtained in the fractionation process by HPLC, bioactive molecules, or corn trypsin inhibitor (CTI), a selective inhibitor of FXIIa, as a positive control for 10 min before adding Spectrozyme FXIIa (0.2 μM) (American Diagnostica Inc.) or adding 100 μL of 0.3 mM HD-Phe-Pro-Arg-p-aranitroanilide (S2302) (DiaPharma; Franklin, OH, USA).

### 4.8. FXII Autoactivation Assays

FXII (0.2 μM) was incubated with varying concentrations of tea water extract, dextran sulfate, or buffer for 1 h before adding S2302 (0.3 mM). To determine if the tea water extract could block autoactivation of FXII, 10 μg tea water extract or buffer was pre-incubated with FXII for 5 min prior to the addition of dextran sulfate (1 μg/mL). The mixture was incubated for 1 h before adding Spectrozyme FXIIa (0.2 mM).

### 4.9. FXIIa-Mediated FXI Activation

Tea water extract (10 μg/μL) or buffer was incubated with FXIIa (5 nM) for 5 min before adding FXI (25 nM). At timed intervals, samples of the reaction were removed, FXIIa activity was quenched by the addition of 1 μM CTI, and FXIa activity was assayed by the addition of 1 mM S2366. The amount of FXIa formed was determined by extrapolating from a standard curve using purified FXIa.

### 4.10. FXIIa-Mediated Prekallikrein Activation

Chromatographic fractions (F18 and F19) of tea water extract (10 μg/mL) or buffer were incubated with FXIIa (4 nM) for 5 min before adding prekallikrein (20 nM). After 30 min, samples of the reaction were removed and FXIIa activity was quenched by the addition of 100 μg/mL CTI. Kallikrein activity was assayed by the addition of 0.25 mM Pefachrome PK, a highly selective chromogenic substrate for plasma kallikrein. The amount of kallikrein formed was determined by extrapolating from a standard curve prepared using purified kallikrein.

### 4.11. Bradykinin Determination

FXIIa (4 nM) was incubated in the absence or presence of tea water extract (10 μg/μL) for 5 min before the mixture of prekallikrein (100 nM)/high-molecular-weight kininogen (100 nM, HK/PK) was added and incubated for 60 min. Afterward, the supernatants were collected and either frozen at −70 °C or immediately deproteinized with trichloroacetic acid. BK in the samples was determined using a commercial kit (MARKIT BK, Dainippon Pharmaceutical, Osaka, Japan), performed according to the manufacturer′s instructions.

### 4.12. MTT Assay

The cytotoxicity of tested compounds and herb extracts were assessed by thiazolyl blue tetrazolium bromide (MTT) colorimetric assay, as previously described. HPAECs (2.5 × 10^4^ cells/well) were seeded in 96-well culture plates and grown for 24 h at 37 °C. Next, 100 μL of fresh medium containing serial dilution of the tea water extracts or arjunglucoside II was added into each well and incubated for 24 h. All extracts and arjunglucoside II were removed and 100 μL 0.5 mg/mL MTT solution was added into each well. After 2 h of incubation, MTT was removed and 100 μL DMSO was added. The plate was shaken on an orbital shaker in dark for 15 min at room temperature. The absorbance was read at 570 nm using a microplate reader with background subtraction at 690 nm. Experiments were repeated three times. Each group had three wells. Data are presented as the mean ± standard deviation (SD).

### 4.13. In Vitro Wound Healing Assay

HPAECs (4 × 10^5^ cells/well) were seeded in 96-well plates to grow in a monolayer for 24 h at 37 °C. Following the incubation, a sterile 20–200 μL pipette tip was held vertically to scratch a cross in each well. The detached cells were removed by washing with 200 μL of pre-warmed PBS twice. Then, 100 μL of fresh medium containing activated FXIIa (240 nM) in the absence or presence of serial dilution of tea water extract was added afterward and incubated for 24 h at 37 °C. Prior to the image acquisition, the microtiter plate was washed with 200 μL of pre-warmed PBS one time. Then, the pre-warmed medium was added again and pictures were taken. The percentage of open wound area was plotted over time for each tea water extract concentration and compared to that of the control. Data are presented as mean ± SD. Three to 5 replicates were included in the analysis and significance was considered at *p* < 0.05.

### 4.14. Molecular Docking Studies

Molecular docking studies were performed using the GOLD software package, version 5.4.1 (Cambridge Crystallographic Data Center, Cambridge, UK) on the crystal structure of the deglycosylated catalytic domain of factor XII (PDB: 4XDE). The active site was defined as a sphere enclosing residues within 10 Å around the ligand. The 3D structure of arjunglucoside II was built using Chem3D (version 15.1) and was energy minimized using an MM2 force field. The energy-minimized structure was docked into both the active site and the glycosylated site of the catalytic domain of FXII, and scored using ChemPLP fitness function. All poses generated by the program were visualized; however, the pose with the highest fitness score was used for elucidating the binding characteristics. Pymol (version 1.3) was used for generating images and superposing with the crystal structure of the glycosylated catalytic domain of factor XII (cyan, PDB: 4XE4).

### 4.15. Statistical Analysis

The mean ± SEMs from the bioassays of plasma kallikrein, FXlla, FXIa, and kallikrein formed on HPAECs were analyzed with nonlinear regression in GraphPad Prism 6.0. GraphPad software, Inc., USA. Experiments were performed at least three times in duplicate or triplicate. Data were analyzed using one-way analysis of variance (ANOVA). For all comparisons, statistical significance was defined as * *p* ≤ 0.05, ** *p* ≤ 0.01, or *** *p* ≤ 0.001.

## 5. Conclusions

This study investigated the roles of the bioactive components in tea, in biological processes related to BK-induced angioedema, activated FXII-induced cell migration, kallikrein-induced BK release from high-molecular-weight kininogen, and, in particular, the human plasma kinin-forming enzyme dynamics. In conclusion, we found the following: (1) Tea is effective in inhibiting activated FXII and kallikrein, whereas the major polyphenols of tea did not inhibit these enzymes. Tea is effective in inhibiting activated FXII-induced cell migration; (2) tea inhibited the release of BK from high-molecular-weight kininogen by activated FXII-mediated kallikrein production; (3) among the major bioactive compounds of Terminalia, arjunglucoside II inhibited activated FXII. Our study emphasizes the link between the bioactive components of tea and the inhibition of FXII- and kallikrein-dependent pathways. More studies at the molecular level are necessary to identify and characterize the bioactive components in tea that influence the plasma kinin-forming enzyme dynamics.

## Data Availability

Not applicable.

## Sample Availability

Samples of the compounds are not available from the authors.
